# Angiotensin II—Real-Life Use and Literature Review

**DOI:** 10.3390/medicina60091483

**Published:** 2024-09-11

**Authors:** Andreja Möller Petrun, Andrej Markota

**Affiliations:** 1 Department of Anaesthesiology, Intensive Therapy and Pain Management, University Medical Centre Maribor, 2000 Maribor, Slovenia; andreja.moellerpetrun@ukc-mb.si; 2 Faculty of Medicine, University of Maribor, 2000 Maribor, Slovenia; 3 Medical Intensive Care Unit, University Medical Centre Maribor, 2000 Maribor, Slovenia

**Keywords:** angiotensin II, vasoplegia, hypotension, distributive shock, immunomodulation

## Abstract

Angiotensin II is a recently introduced vasopressor, which has been available since 2017. The novelty and the relatively high cost of angiotensin II currently limit its broader application. It induces vasoconstriction by activating the renin–angiotensin–aldosterone system and is currently the sole vasopressor functioning through this pathway. Beyond vasoconstriction, angiotensin II also affects various other physiological processes. Current evidence supports its use in managing vasoplegic and cardiogenic shock in patients who are unresponsive to catecholamines and vasopressin. However, due to limited data, the optimal timing for initiating therapy with angiotensin II, strategies for combining it with other vasopressors, and strategies for its discontinuation remain unclear. Ongoing and planned studies aim to address some of these uncertainties. This article reviews the physiological and pathophysiological effects of angiotensin II, describes its pharmacology, and provides a narrative review of the current literature.

## 1. Introduction

Shock is a life-threatening condition that results in an insufficient delivery of oxygen and nutrients to tissues, leading to multiorgan failure or even death [1]. Septic shock is the most common type of shock among critically ill patients, regardless of the patient population in a particular intensive care unit. The guidelines for treating sepsis and septic shock are based on large randomized trials [2]. A major limitation of these guidelines is that they are designed for an average population and may not account for the genetic and other individual peculiarities of each patient. 

In the perioperative intensive care unit, hemorrhagic and cardiogenic shock, as well as vasoplegia associated with the systemic inflammatory response after major surgeries, are also encountered, particularly following heart operations involving extracorporeal blood circulation. Recommendations for managing these types of shock are less definitive. Increasingly, the literature emphasizes the need to tailor medical procedures and treatments to individual patient characteristics—an approach known as personalized medicine [1,3]. 

Angiotensin II (ATII) is a new vasopressor that has shown effectiveness in patients with distributive and cardiogenic shock states, and which could potentially be used for specific individual patients [4,5]. This text will discuss the known physiological and pathophysiological effects of ATII, its basic pharmacological characteristics, and review the latest literature on its clinical use. 

## 2. Renin–Angiotensin–Aldosterone System

In recent years, it has become increasingly evident that the renin–angiotensin–aldosterone system (RAAS) is significantly more complex and influenced by numerous endogenous and exogenous factors, as opposed to the simpler models described in classic physiology textbooks [6,7]. The renin–angiotensin system (RAAS) operates at the endocrine, paracrine, and intracrine levels [6,8]. The classical and non-classical RAASs are two opposing systems that maintain balance in a healthy organism [9]. Detailed descriptions of these systems can be found in the review articles by Bitker et al. and Garcia et al. [6,7]. 

In the classical RAAS, angiotensin-converting enzyme (ACE) and ATII play crucial roles. In contrast, the non-classical RAAS involves angiotensin-converting enzyme 2 (ACE2) and angiotensin (1–7) [6]. ACE2 is also implicated in the entry of the SARS-CoV-2 virus into pneumocytes and intestinal cells, playing a significant role in adult acute respiratory distress syndrome (ARDS), though a detailed discussion of this area is beyond the scope of this paper [6,7,10]. 

The circulating RAAS, in conjunction with catecholamines and vasopressin, is vital for regulating blood pressure and maintaining water and electrolyte homeostasis, the latter being dependent on aldosterone. At the paracrine and intracrine levels, RAAS modulates vascular permeability, influences inflammatory processes, and affects cell growth, differentiation, migration, and apoptosis [11,12]. 

The starting molecule for both the classical and non-classical RAAS is angiotensinogen, a glycosylated protein produced in the liver. Angiotensinogen itself is inactive, with its blood concentration remaining relatively constant. Renin, an enzyme released from pericytes near afferent arterioles and the juxtaglomerular apparatus in the kidneys, converts angiotensinogen to angiotensin I, a biologically inactive decapeptide. The release of renin is triggered by lowered arterial pressure, dehydration, hypoxic conditions, and activation of the sympathetic nervous system. Renin activity is a limiting factor in the functioning of the RAAS [13,14,15]. Additionally, renin release is regulated by ATII concentration through a negative feedback loop [6].

## 3. Classical RAAS

**Figure 1:**
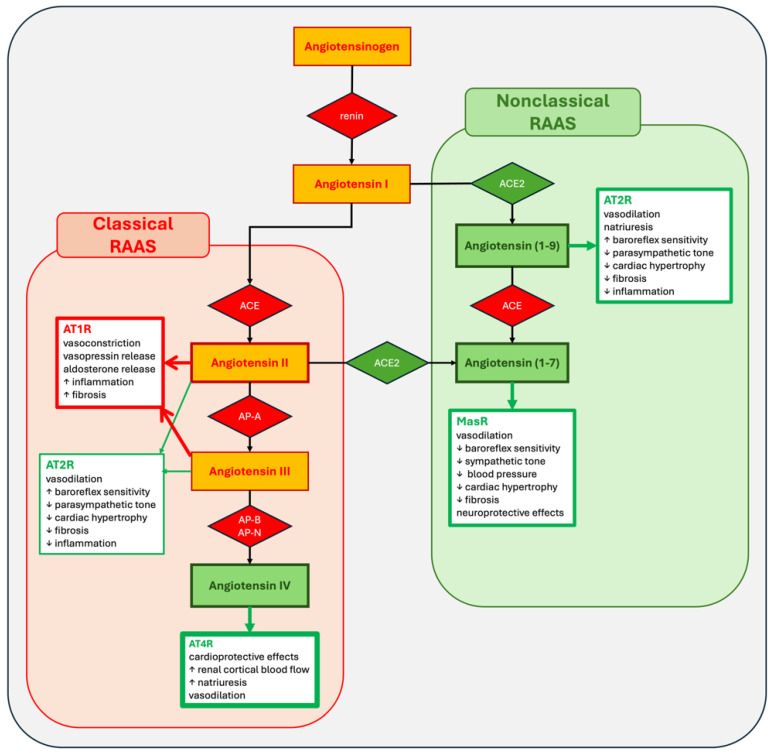
Simplified diagram of the classical and nonclassical RAAS. Legend: RAAS—renin angiotensin-aldosterone system; ACE—angiotensin-converting enzyme; ACE2—angiotensin-converting enzyme type 2; APA—aminopeptidase A; AP-B—aminopeptidase B; AP-N—aminopeptidase N; AT1R—angiotensin II receptor type 1; AT2R—angiotensin II receptor type 2; AT4R—angiotensin II receptor type 4; MasR—Mas receptor.

In the lungs, ACE converts angiotensin I to ATII (Figure 1) [7]. Among other physiological effects this also serves as the main target for ACE inhibitors [16]. ATII is a potent vasopressor, but it is rapidly degraded by various enzymes in the plasma into angiotensin III, angiotensin IV, and angiotensin (1–7). Similar to ATII, angiotensin III is a potent vasopressor that binds to angiotensin type 1 and type 2 receptors (AT1R and AT2R). It also stimulates the release of aldosterone and promotes thirst and appetite for salty food [6,7,15,16]. The primary role of angiotensin IV is to decrease sodium and water reabsorption in the kidneys and activate nitric oxide synthase, thereby opposing the action of ATII [6,17]. Angiotensin IV binds to AT4 receptors (AT4R), which are mainly located in the brain, where they likely regulate cognitive–sensory and motor functions, and in renal arteries [6,18]. At higher concentrations, angiotensin IV can also bind to AT1R and produce effects similar to ATII [7]. 

ATII exerts its effects primarily by binding to AT1R, but it also binds to AT2R [7,16]. In addition to AT1R and AT2R, other known receptors include AT4R and the MAS receptor [18]. AT1Rs are G-protein-coupled receptors located on peripheral vascular smooth muscle cells, where they mediate the vasopressor effects of ATII, promote the tubular reabsorption of sodium, chloride, and bicarbonate, and stimulate the secretion of potassium and hydrogen ions [15]. ATII induces adrenergic stimulation, increases transcription of endothelin-1, releases aldosterone, and enhances cardiac output by inhibiting vagal tone. One of ATII’s central effects is the release of vasopressin from the supraoptic nucleus via the pituitary gland into the bloodstream [19]. 

ATII also influences cell growth, apoptosis, inflammatory responses, the blood coagulation system, and possibly mitochondrial function [14]. Its role in myocardial remodeling in congestive heart failure is well documented [20]. AT1Rs are also present in the adrenal glands, heart muscle, granulocytes, lymphocytes, and the glomeruli and proximal tubules of the kidneys [6]. Chronic abnormal activation of AT1R can lead to heart muscle hypertrophy, vascular endothelial inflammation, atherosclerosis, oxidative stress, cancer cell development, autoantibody production, and malignant hypertension [21]. AT1Rs are the primary targets of sartans, a class of antihypertensive drugs [22]. 

## 4. Nonclassical RAAS

Angiotensin (1–7) is the primary molecule in the nonclassical renin–angiotensin system (RAAS) (6). It can be produced through two pathways: ACE2 cleaves angiotensin I to angiotensin [1,2,3,4,5,6,7,8,9], which is then converted to angiotensin (1–7) by ACE, or alternatively ACE2 cleaves ATII directly to angiotensin (1–7) (Figure 1). The receptor for angiotensin (1–7) is MAS, a proto-oncogene [7,18]. The binding of angiotensin (1–7) to MAS receptors enhances the production of arachidonic acid and nitric oxide synthase. The activation of MAS receptors also reduces the sensitivity of the baroreceptor reflex, the tone of the sympathetic nervous system, blood pressure, myocardial hypertrophy, and fibrosis [7]. MAS receptors are primarily located in the brain, where they are involved in cardiovascular regulation, neuroprotection, and possibly cognitive functions [23]. MAS is also present in the renal epithelium, heart, and vascular endothelium [4,6,18]. 

Angiotensin (1–9) binds to AT2 receptors (AT2R), which mediate effects that generally oppose those of AT1 receptors (AT1R). AT2Rs are found in the endothelium, kidneys, adrenal glands, heart muscle, and brain [6]. Angiotensin (1–9) has cardioprotective and antifibrotic roles. AT2Rs are also likely involved in pain modulation, the development of the central nervous system and urinary tract, antidiuretic and antinatriuretic functions, and certain metabolic disorders [18]. 

There is some evidence that, during sepsis and shock states, which are characterized by vasodilation, activation of the nonclassical RAAS occurs. This activation may open new therapeutic possibilities, particularly with the use of exogenous angiotensin (1–7) and ACE2 [7].

## 5. Angiotensin II as a Vasopressor

In critically ill patients with vasodilatory shock, the activation of the classical RAAS is pathophysiologically altered, disrupting the physiological response aimed at maintaining homeostasis [6,24]. In septic patients, elevated levels of renin and angiotensin I are correlated with disease severity, and the angiotensin II (ATII) signaling pathway is impaired [25,26]. This results in decreased ACE activity, increasing the angiotensin I/ATII ratio, which is associated with higher mortality [24]. A similar phenomenon occurs in patients undergoing extracorporeal circulation during heart surgery, as blood bypasses the lungs, reducing the conversion of angiotensin I to ATII. This ATII deficiency contributes to vasoplegia, a condition frequently observed in these patients, albeit temporarily [27]. Sepsis models also show reduced expression of AT1 receptors (AT1R) in blood vessels and kidneys [7,28]. 

Exogenous ATII infusion has shown potential benefits in these patients by decreasing the angiotensin I/ATII ratio, thereby reducing the need for vasopressors such as norepinephrine and vasopressin [29,30,31]. ATII induces direct vasoconstriction through its action on smooth muscle cells in the vessel walls, enhances sympathetic tone, and stimulates the endogenous release of vasopressin and catecholamines [30]. Additionally, ATII exhibits favorable immunomodulatory effects, particularly in the initial phase of septic shock, which are discussed further below. Despite its use as a vasopressor in numerous clinical studies over several decades, ATII was only approved for clinical use by the US Food and Drug Administration (FDA) in 2017 and by the European Medicines Agency (EMA) in 2019 [32,33,34]. 

In a 2009 sheep model of Gram-negative septic shock, Wan et al. demonstrated that acute renal failure in sepsis is likely related to vasodilation of efferent arterioles. They showed that ATII infusion improves renal function and systemic blood pressure [35]. ATII is also believed to reduce intrarenal inflammation and apoptosis [36]. The first recent pilot study on ATII use in treating distributive shock in humans, the ATHOS trial, was published in 2014 (Table 1). It found that ATII reduced the need for catecholamines and vasopressin in patients requiring multiple vasopressors due to distributive shock [37]. This was followed by the double-blind multicenter ATHOS-3 study, which found that ATII significantly increased the mean arterial pressure in patients requiring norepinephrine at doses equivalent to >0.2 μg/kg/min (Table 1) [30]. The odds ratio for achieving a higher mean arterial pressure was eight times greater for ATII compared to a placebo. A post hoc analysis of a subgroup of ATHOS-3 patients who required renal replacement therapy due to acute renal failure showed that ATII had a beneficial effect compared to placebo [31]. Moreover, patients with elevated plasma renin levels who received ATII had improved survival rates [26]. 

In a post hoc analysis of ATHOS-3, Wieruszewski et al. determined that patients who received ATII in combination with lower doses of norepinephrine and vasopressin had better clinical outcomes compared to those who received higher doses of these vasopressors [38]. Similarly, Smith et al. reported comparable results in a retrospective multicenter study on ATII use in shock treatment [39]. See et al. conducted a pilot study involving 40 ICU patients who received ATII as the primary vasopressor and compared them with 80 patients receiving standard vasopressors. They observed better survival rates, less acute kidney injury in patients previously on ACE inhibitors, and lower serum troponin levels in the ATII group, with a similarly low thrombosis incidence in both groups (Table 1) [40]. 

Wieruszewski et al. published the largest post-marketing study on ATII use in shock patients in 2021. They observed that 67% of the 270 patients had a favorable hemodynamic response to ATII infusion, with better survival rates in responsive patients. Patients with lower serum lactate concentrations and those previously on vasopressin were more responsive to ATII infusion (Table 1) [41]. 

Regarding ATII use for vasoplegia during and after heart surgery with extracorporeal circulation, two studies have been conducted, with more ongoing. These studies concluded that ATII use during and after such operations is safe, achieving target mean arterial pressure values, and resulting in lower plasma renin concentrations compared to patients receiving only catecholamine vasopressors. Moreover, ICU treatment duration was shorter in patients who received ATII [42,43]. 

Concerns about ATII’s potential negative impact on cardiac function have led to the exclusion of patients with reduced ejection fraction from most studies. However, a recently published observational study on ATII use in patients requiring extracorporeal mechanical support due to cardiogenic shock concluded that ATII infusion was safe, with favorable hemodynamic effects and no deterioration of cardiac function [5].

**Table 1:** Overview of select clinical studies on ATII.

Authors [Reference]	Type of Study	Number of Patients	Main Findings
Chawla et al. [37]	prospective randomized pilot trial	20	Infusion of ATII at 20 ng/kg/min resulted in a reduction in NA from 19.8 ± 11.7 to 7.4 ± 12.4 mcg/min; infusion of placebo resulted in reduction in NA from 30.3 ± 20.4 to 27.6 ± 29.3 mcg/min.
Khanna et al. [30]	prospective randomized controlled trial	321	69.9% of patients reached the primary endpoint (MAP increase ≥10 mmHg or to ≥75 mmHg) in the ATII group, compared to 23.4% of patients in the placebo group; 28-day mortality was 46% in ATII group and 54% in the placebo group.
Smith et al. [39]	retrospective observational study	162	Reduction in NA-equivalent dose of vasopressors by 0.16 mcg/kg/min and increase in MAP by 9.3 mmHg between 0 and 3 h after the initiation of ATII.
See et al. [40]	prospective observational study	120	Lower ICU mortality (10% vs. 26%) in patients who received ATII as primary vasopressor compared to NA as primary vasopressor, with similar peak creatinine levels (128 vs. 126 mcmol/L) and incidence of acute kidney injury (70% vs. 74%).
Wieruszewski et al. [41]	retrospective observational study	270	67% of patients achieved primary endpoint (MAP ≥ 65 and identical or reduced total vasopressor dose 3h after initiation of ATII); in patients who achieved primary endpoint, the MAP increased by 10.3 mmHg and the NA-equivalent dose of vasopressors decreased by 0.2 mcg/kg/min compared to patients who did not reach the primary endpoint (MAP increased by 1.6 mmHg and NA-equivalent dose of vasopressors increased by 0.04 mcg/kg/min).

Legend: ATII—angiotensin II; ICU—intensive care unit; MAP—mean arterial pressure; NA—noradrenaline.

## 6. Immunomodulatory Effects of Angiotensin II

Activation of both the classical and nonclassical RAAS impacts both innate and adaptive immunity [6]. These systems regulate inflammation, cell proliferation, fibrogenesis, and apoptosis. ATII, via the classical RAAS, stimulates neutrophil and macrophage chemotaxis, the production of reactive oxygen species, and the secretion of proinflammatory cytokines. Additionally, ATII influences lymphocyte activation, inhibiting CD8+ T lymphocyte activation, a process in which ACE also plays a crucial role [12]. 

Experimental research using a mouse model demonstrated that ATII has complex immunomodulatory effects within the RAAS and acts as a potent opsonin, accelerating bacterial clearance from the body [44,45]. Through AT1 receptors (AT1R) on leukocytes, ATII enhances the innate myeloid immune response to severe systemic infections, thereby aiding bacterial defense [46,47]. 

In contrast, the activation of the nonclassical RAAS exerts immunoprotective effects on tissues during sepsis, which are physiologically opposite to those of the classical RAAS [6].

Norepinephrine, the most commonly used vasopressor and currently the first-line treatment for hypotensive patients with sepsis, has well-documented immunosuppressive effects. It negatively affects proinflammatory cytokines compared to vasopressin and likely contributes significantly to the immunoparalysis associated with septic shock [48]. Given the limited data on ATII, it remains a second- or third-line vasopressor in septic shock treatment [2]. However, if further clinical research substantiates the beneficial effects of ATII on the immune response, ATII may assume a more prominent role in treating vasoplegia, particularly within the framework of personalized medicine and the specific phases of septic shock. 

## 7. Adverse Effects Associated with Angiotensin II

In 2017, prior to its approval for clinical use, Busse et al. conducted a systematic review of the existing literature on the adverse effects of ATII and concluded that its safety profile was acceptable [32]. In over 31,000 patients who received ATII as a vasopressor in clinical trials, side effects were relatively rare. The most common adverse effects included headache, chest pain, and orthostatic symptoms. The most serious side effects, observed in a few individual cases, were exacerbation of asthma (bronchoconstriction) and worsening of left-sided heart failure in patients with pre-existing left-sided heart failure. There was also one reported case of intracerebral hemorrhage with a fatal outcome. 

The drug information provided by the manufacturer, the FDA, and the EMA also mentions an increased risk of thrombosis associated with ATII use [33,34]. The mechanism behind ATII-induced thrombosis is not well understood, but it is thought to involve the immunomodulatory effects of ATII (primarily mediated through IL-6) and its impact on the endothelium of small vessels [49]. ATII also influences the expression of PAI-1 (plasminogen activator inhibitor 1) [50]. In vitro studies suggest an increased risk for both arterial and deep vein thrombosis. According to the manufacturer, the overall risk of thrombosis is 12.9% with ATII compared to 5.1% with a placebo, and the risk of deep vein thrombosis is 4.3% with ATII compared to 0% with a placebo. However, these percentages do not differentiate between clinically significant and insignificant thromboses [33]. 

Clinical research data do not strongly support an increased risk of thrombosis with ATII use. The authors of the original ATHOS-3 trial reported no clinically significant difference in the incidence of deep vein thrombosis between ATII (1.8%) and placebo (0%) [30]. Similarly, a pilot study investigating ATII as a primary vasopressor in critically ill patients found no major occurrences of thrombosis [40]. In the largest post-marketing study of ATII for the treatment of shock, thrombosis occurred in 4 out of 270 patients [41]. A retrospective study by Smith et al. reported a low incidence of deep vein thrombosis (3.1%) with only one case being clinically significant, despite only 75.4% of patients receiving antithrombotic prophylaxis [39]. 

The challenge in determining the exact cause of ATII-induced thrombosis is compounded by the underlying conditions, such as sepsis, that necessitate vasopressor infusion. It is difficult to discern whether thromboses are due to the underlying disease or the ATII infusion itself. 

Other adverse effects of ATII include thrombocytopenia, tachycardia, and ischemia of the intestines and extremities (4.3% with ATII vs 2.5% with placebo) [34]. 

## 8. Dosing 

ATII is available exclusively for intravenous administration. In Europe, it is marketed in 2 mL vials containing 2.5 mg/mL, while in North America, 1 mL vials of the same concentration are also available. The medication is diluted in either 500 mL or 250 mL of saline solution, depending on the patient’s volume status. Administration via a central venous catheter is recommended. The initial dose is typically 20 ng/kg/min (based on the patient’s ideal body weight). This dose can be titrated every 5 min in increments of 5–15 ng/kg/min to achieve the desired mean arterial pressure, with a maximum dose of 80 ng/kg/min within the first three hours. The maximum recommended maintenance dose after this period is 40 ng/kg/min. It is advised to use the lowest effective dose that achieves the target mean arterial pressure. 

Response to ATII has usually been described as an increase in mean arterial blood pressure of around 10 mmHg after the initiation of ATII infusion [5,30,32,37,41]. While this description makes sense for research purposes, in a real-life setting it can be less useful. First, because of a multitude of other factors affecting the mean arterial pressure simultaneously (other vasopressors, fluids, etiological treatment of the underlying pathology, such as antibiotics and hemostasis, mechanical ventilation, etc.) [2], and second, because of the open-ended nature of such a definition of responsiveness (i.e., if the patient does not respond to 20 ng/kg/min of ATII, the dose is increased to 40 ng/kg/min, and in the case of no response to 60, 80, or even higher doses), which exposes the patient to significant fluid loading because of the infusion of ATII [33,34], incurs significant expenses to the institution, and opens questions about strategies for weaning the patient off ATII.

According to authors’ experience in the use of ATII, the effects of ATII in responsive patients are observed within 2–5 min, consistent with the reports in the literature. In such patients, it is typically possible to reduce or discontinue norepinephrine and vasopressin doses within minutes to hours. A response is achieved in around two-thirds of patients, and patients mostly respond to doses not exceeding 40 ng/kg/min. Responders to higher doses are rare and in our institution we have mostly refrained from using ATII in higher doses. In cases where it is given in higher doses, this is performed on a “test vial” principle, i.e., if the patient responds the infusion will be continued until reevaluation, and if the patient does not respond the infusion will be either discontinued or continued at 40 ng/kg/min. Re-evaluation is conducted with a complete clinical picture in mind in order to try to determine the most realistic patient outcome. Once hemodynamic stability is achieved, the ATII dose is gradually reduced (over several hours) in steps of 5–15 ng/kg/min, as long as the mean arterial pressure remains stable. The literature indicates that ATII infusion is usually administered for up to 48 h, which aligns with our hospital’s experience.

Our institutional guidelines allow for the initiation of ATII in patients with distributive shock who remain hypotensive despite a norepinephrine dose of at least 0.3 mcg/kg/min and infusion of vasopressin. Based on the literature, it may be reasonable to consider ATII at norepinephrine dose equivalents of 0.2 mcg/kg/min or lower in certain patients. 

As per institutional guidelines, blood is withdrawn for renin determination before initiating ATII infusion; however, there are no institutionally set cut-off values precluding the clinician from using ATII in patients with low renin. Additional renin determination is performed after 24–48h of ATII infusion to help guide clinicians in deciding if the patient is responding to ATII. Renin is a novel marker of disease severity in critically ill patients regardless of therapy with ATII [51] and it could also predict hemodynamic responses to ATII [26]; however, because of its limited availability we think that its role in predicting hemodynamic response is limited compared to that in outcome prediction. In our institution renin is available from 8 a.m. to 2 p.m. Monday to Friday, and samples are stored (at −85 °C) and processed on the next possible morning if blood is withdrawn outside this time. We acknowledge that there are no widely agreed cut-off levels of renin, however, we think that it has been sufficiently well established that persistent hyperreninemia is associated with poor outcomes, and this could be one of factors considered during patient reevaluation [51,52,53].

The mechanism behind vasoplegia during septic shock (which is the most common form of distributive shock in patients who receive ATII in the literature and in our experience) is multifactorial; endotoxemia and proinflammatory cytokines cause a systemic down-regulation of catecholamine and ATII receptors; vasopressin is rapidly depleted during the first few hours of shock; and endothelial injury and reduced ACE activity lead to reduced endogenous ATII levels, all of which possibly favor early initiation of multi-pronged vasopressor therapy [2,4,27]. The authors suggest that ATII is not used as “rescue” therapy for patients who require excessively high doses of vasopressors, but in the earlier phases of septic shock. Our institutional guidelines for the use of ATII (patients who are hypotensive despite noradrenaline at 0.3 mcg/kg/min and vasopressin) are an attempt at balancing the cost and novelty of ATII against the presumed benefit of early initiation. ATII use in individuals under 18 is not approved due to insufficient data. There is also no information on potential drug interactions. Specific pharmacokinetic studies have not been conducted, but physiologically, ATII’s plasma half-life is less than 1 min, allowing for precise titration. 

## 9. Conclusions

Angiotensin II is a recently rediscovered drug for treating shock states with a unique mechanism of action affecting catecholamine and arginine-vasopressin systems. The literature supports its efficacy as a vasopressor in appropriately selected patients, with beneficial effects on kidney function and bacterial clearance in early septic shock. Despite initial concerns, its use appears safe even in cardiogenic shock. The optimal timing for initiating ATII infusion remains unclear, but earlier use is generally supported. In some cases, ATII may be considered as a primary vasopressor due to its mechanism of action. The main barriers to widespread clinical use are the novelty of ATII and its relatively high cost.
